# Prediction of ineffective elective cardioversion of atrial fibrillation: a retrospective multi-center patient cohort study

**DOI:** 10.1186/s12872-017-0470-0

**Published:** 2017-01-18

**Authors:** Tapio Hellman, Tuomas Kiviniemi, Tuija Vasankari, Ilpo Nuotio, Fausto Biancari, Aissa Bah, Juha Hartikainen, Marianne Mäkäräinen, K. E. Juhani Airaksinen

**Affiliations:** 1 Heart Center, Turku University Hospital and University of Turku, Hämeentie 11, PO Box 52, 20521 Turku, Finland; 2 Department of Medicine, Turku University Hospital and University of Turku, Hämeentie 11, PO Box 52, 20521 Turku, Finland; 3 Heart Center, Kuopio University Hospital and University of Eastern Finland, PO Box 100, Kuopio, 70029 Finland; 4 Department of Surgery, Oulu University Hospital, Kajaanintie 50, PO Box 10, 90029 OYS Oulu, Finland

**Keywords:** Atrial fibrillation, Cardioversion, Recurrence

## Abstract

**Background:**

Elective cardioversion (ECV) of atrial fibrillation (AF) is a standard procedure to restore sinus rhythm. However, predictors for ineffective ECV (failure of ECV or recurrence of AF within 30 days) are unknown.

**Methods:**

We investigated 1998 ECVs performed for AF lasting >48 h in 1,342 patients in a retrospective multi-center study. Follow-up data were collected from 30 days after ECV.

**Results:**

Median number of cardioversions was one per patient with a range of 1–10. Altogether 303/1998 (15.2%) ECVs failed. Long (>5 years) AF history and over 30 days duration of the index AF episode were independent predictors for ECV failure and low (<60/min) ventricular rate of AF predicted success of ECV. In patients with successful ECVs an early recurrence of AF was detected in 549 (32.4%) cases. Female gender, high (>60/min) ventricular rate, renal failure and antiarrhythmic agents at discharge were the independent predictors for recurrence. In total ECV was ineffective in 852 (42.6%) cases. Female gender (OR 1.44, CI95% 1.15–1.80, *p* < 0.01), young (<65 years) age (OR 1.31, CI95% 1.07–1.62, *p* = 0.01), ventricular rate >60/min (OR 1.92, CI95% 1.08–3.41, *p* = 0.03), antiarrhythmic medication at discharge (OR 1.48, CI95% 1.14–1.93, *p* < 0.01) and low (<60/ml/min) estimated glomerular filtration rate (OR 1.59, CI95% 1.08–2.33, *p* = 0.02) were predictors of ineffective ECV.

**Conclusions:**

Female gender, use of antiarrhythmic drug therapy and renal failure predicted both recurrence of AF and the composite end point. For the first time in a large real-life study several clinical predictors for clinically ineffective ECV were identified.

## Background

Atrial fibrillation (AF) is the most common cardiac arrhythmia and affects almost 10% of patients >80 years of age [1]. Elective cardioversion (ECV) is an essential part of rhythm control strategy of AF. Rhythm control strategy does not, however, offer prognostic benefit over the rate control in terms of mortality or quality of life [2]. Furthermore, approximately 10–35% of ECVs fail and recurrences of AF after successful ECV are common causing extra burden and costs for the health care system. Numerous small studies have tried to explore predictors for failure of ECV and recurrence of AF, but without consistent results [3–6]. We sought to explore the rate and predictors of ineffective ECV defined as failed ECV or early (<30 days) recurrence of AF in a large-scale multi-center patient cohort in contemporary practice.

## Methods

The FinCV2 study ([http://www.ClinicalTrials.gov], identifier NCT02850679) is part of a wider protocol in progress to assess thrombotic and bleeding complications of AF in Finland [7, 8].

In this multi-center retrospective study data was gathered from patient charts of two university hospitals and two regional hospitals in Finland. Initial screening was performed using the ICD-10 code for AF (I48) and the NCSP (Nordic Classification of Surgical Procedures) procedure code for cardioversion (TFP20). All patients with AF lasting > 48 h undergoing ECV within the study period were eligible during the time period of 2003 – 2014 in Turku University Hospital and two regional hospitals and 2013 – 2015 in Kuopio University Hospital.

Overall 2,373 patients with a history of both AF and undergoing cardioversion were initially screened. Patients with AF (duration >48 h) and subsequent ECVs were then manually identified and included resulting in the final study group of 1342 patients and 1998 ECVs.

A structured electronic case report form was used in the manual data collection. Data consisting of patient history, medication, AF disease and ECV characteristics including success rate were recorded. After the ECV patient records of the following 30 days were examined and data on all cerebrovascular events, systemic emboli, bleeds, AF recurrences and mortality were collected.

All AFs were confirmed by ECG and the clinician performing the cardioversion. The overall duration of AF disease was divided into six groups: 31–90 days, 90–180 days, 180 days–1 year, 1–2 years, 2–5 years and >5 years. Correspondingly, the duration of current AF episode was divided in <30 days, 30–60 days, 61–90 days, 91–120 days, 121–180 days and > 180 days. Estimated glomerular filtration rate (eGFR) was calculated using simplified Modification of Diet in Renal Disease (MDRD) formula.

ECVs were performed by an internist or a cardiologist according to the current guidelines under general anesthesia. Blood pressure and oxygen saturation were monitored. A 12-lead ECG was controlled before and after ECV. Paddles or pads were positioned in antero-posterior or antero-lateral configuration. The energy ranged from 70 to 200J with biphasic defibrillator devices and from 70 to 360J with monophasic devices. ECVs were performed by biphasic defibrillators after 2004.

The primary end point was ineffective ECV defined as the composite of unsuccessful ECV or recurrence of AF within 30 days follow-up after the index ECV. The restoration and maintenance of sinus rhythm after ECV until discharge was defined as successful ECV. Development of AF within 30 days after the index ECV confirmed by ECG or pacemaker log was defined as an AF recurrence.

Normally distributed continuous variables were reported as mean ± SD whereas skewed continuous variables were denoted as median [inter-quartile range (IQR)]. Normality in continuous covariates was tested with Kolmogorov-Smirnov and Shapiro-Wilk tests. Categorical variables were reported with absolute and relative (percentage) frequencies. The unpaired t-test or Mann-Whitney test was used to compare continuous variables and Pearson x^2^ or Fisher’s exact test to compare categorical variables in the study subgroups, as appropriate. Logistic regression hazard model was used to identify the independent predictors of ECV failure, recurrence of AF and ECV failure (a combination of the above covariates). Baseline variables correlating at *p* < 0.10 level with the dependent covariate in univariate models were entered in the logistic regression analysis. Correlations at level *p* < 0.05 were considered statistically significant. All tests were two-sided. IBM SPSS Statistics software version 22.0 was used to perform all analyses.

## Results

The median number of cardioversions was one per patient with a range of 1–10. ECV failed in 303 (15.2%) cases. Patients with failed ECV had significantly longer history of AF, longer duration of current AF episode and higher ventricular rate. Patients with lower (<60 ml/min) eGFR had lower success rates. In multivariate regression analysis prolonged (>5 years) AF history (OR 1.56, CI95% 1.03–2.38, *p* = 0.04), >30 days duration of the index AF episode (OR 1.63, CI95% 1.05–2.55, *p* = 0.03) and higher (>60/min) ventricular rate (OR 12.74, CI95% 1.75–92.91, *p* = 0.01) were the independent predictors for failed ECV.

During the 30 days follow-up AF recurrence was diagnosed in 549 (32.4%) of those patients with initially successful ECV. The median time to recurrence of AF was 8.0 (IQR 13.0) days. Young age (<65 years), female gender, high (>60/min) ventricular rate, short duration of the index AF episode, congestive heart failure, history of kidney disease, low (<60 ml/min) eGFR, diabetes and antiarrhythmic medication at discharge were significant univariate predictors of AF recurrence. In multivariate analysis the significant predictors for recurrence of AF were female gender (OR 1.39, CI95% 1.09–1.78, *p* = 0.01), history of kidney disease (OR 1.59, CI95% 1.00–2.50, *p* = 0.05), low (<60 ml/min) eGFR (OR 1.97, CI95% 1.29–2.99, *p* < 0.01) and antiarrhythmic medications at discharge (OR 1.72, CI95% 1.29–2.29, *p* < 0.01). The type of antiarrhythmic agent at discharge did not affect the sustainability of sinus rhythm.

Taken together, the primary outcome of ineffective ECV occurred in 852 (42.6%) cases. Baseline characteristics of patients with and without ineffective ECV are listed in Table 1. Female gender (OR 1.44, CI95% 1.15–1.80, *p* < 0.01), age <65 years (OR 1.31, CI95% 1.07–1.62, *p* = 0.01), ventricular rate >60/min (OR 1.92, CI95% 1.08–3.41, *p* = 0.03) and antiarrhythmic medication at discharge (OR 1.48, CI95% 1.14–1.93, *p* < 0.01) were the significant independent predictors in multivariate analysis. When renal failure was included in the model in the 1043 cases with eGFR data, ventricular rate >60/min (OR 2.93, CI95% 1.33–6.47, *p* = 0.01), low (<60 ml/min) eGFR (OR 1.59, CI95% 1.08–2.33, *p* = 0.02) and antiarrhythmic medication at discharge (OR 1.50, CI95% 1.09–2.08, *p* = 0.01) remained as significant predictors.

**Table 1:** Baseline characteristics of patients with effective ECV and ineffective ECV

	Effective ECV (*N* = 1146)	Ineffective ECV (*N* = 852)	*p*
Age mean (median) years	64 (65)	63 (64)	0.01
Age > 75 years	176 (15.4)	117 (13.7)	0.06
Female	284 (24.8)	267 (31.3)	<0.01
First AF episode	528 (46.1)	397 (46.6)	0.82
Prior cardioversion	532 (46.4)	380 (44.6)	0.61
CHA_2_DS_2_-VASc-score ≥2	655 (57.2)	468 (54.9)	0.34
Duration of AF disease ≤1 year	527 (53.2)	374 (51.7)	0.56
Age of index AF episode			
> 180 days^a^	76 (6.6)	65 (7.6)	0.60
< 30 days^a^	215 (18.8)	175 (20.5)	0.60
Ventricular rate (/min)			
< 60/min	45 (4.6)	17 (2.3)	0.01
median	87 (30)	87 (30)	0.37
Left atrial diameter (mm)^b^			
median	46 (9)	46 (8)	0.40
Ejection fraction (%)^c^			
median	54 (17)	53 (17)	0.03
Prior AF ablation	41 (3.6)	72 (8.5)	<0.01
History of heart failure	173 (15.1)	150 (17.6)	0.14
Hypertension	598 (52.2)	451 (52.9)	0.75
History of CKD	59 (5.1)	54 (6.3)	0.28
GFR <50 ml/min^d^	24 (4.1)	32 (7.1)	0.04
Diabetes	171 (14.9)	115 (13.5)	0.40
Prior stroke/TIA	76 (6.6)	55 (6.5)	0.93
Coronary artery disease	179 (15.6)	115 (13.5)	0.20
Pacemaker	105 (9.2)	82 (9.6)	0.76
Medication at discharge			
Beta blocker	946 (83.1)	720 (85.2)	0.22
Digoxin	89 (7.8)	123 (14.6)	<0.01
Verapamil	10 (0.9)	10 (1.2)	0.51
Any antiarrhythmic agent^e^	163 (14.3)	158 (18.8)	0.01
Flecainide	67 (5.9)	44 (5.2)	0.55
Amiodarone	65 (5.7)	79 (9.4)	0.01

Values in parentheses are % or interquartile range, *AF* atrial fibrillation, *CKD* chronic kidney disease, *ECV* elective cardioversion, *GFR* glomerular filtration rate, *TIA* transient ischemic attack

^a^data is missing in 834 (41.7%) cases

^b^data is missing in 1194 (59.8%) cases

^c^data is missing in 1142 (57.2%) cases

^d^data is missing in 955 (47.8%) cases

^e^Antiarrhythmic agents comprised flecainide, amiodarone, propafenone, quinidine or disopyramide and dronedarone

## Discussion

In this retrospective real-life cohort study every sixth ECV failed, AF recurrence was diagnosed in one third of patients with successful ECV and thus, ECV was ineffective in almost half of the patients. Long AF history, more than 30 days duration of the index AF episode and higher ventricular rate predicted failure of ECV. The predictors for the ineffective ECV (composite endpoint of ECV failure and early recurrence) were moderate to severe renal impairment, young age, female gender and antiarrhythmic medication at discharge.

The failure rate of ECV in our study (15.2%) was in line with previous studies [3–6, 9–12]. In prior reports the failure rate of cardioversions performed in AF lasting >48 h has ranged from 9–34% [3, 4, 6, 9, 10, 12]. In accordance with previous studies, longer duration of the index episode of AF predicted failure of ECV [3–6, 9] and our results support the view that it is reasonable to minimize the delay between the diagnosis of AF and ECV. Other previously reported predictors for failure of ECV are increasing body weight [6], low ejection fraction [6] and high body surface area [10] whereas pretreatment with antiarrhythmic agents [3], paroxysmal AF [12], use of biphasic waveform in ECV [12], flutter rhythm and young age [9] have predicted success of ECV.

In agreement with previous reports, AF recurred in one third of the patients after successful ECV [3–5, 9, 11–16]. Predictors for recurrence of AF after ECV have been inconsistent [3, 5, 11, 12, 15–17] and previous attempts to establish a scoring system for predicting recurrence of AF after ECV have failed [17]. Our large patient cohort identified several clinical predictors for recurrence of AF in a real-life setting. Interestingly, history of chronic kidney disease and renal failure were significantly associated with the recurrence of AF and the composite end point of ineffective ECV. The association remained significant regardless of the method to diagnose renal failure (clinical history and/or eGFR). Previously, low eGFR has been associated with recurrence of AF after ECV only in a small study on selected group of patients [18]. Traditionally recurrence rate of AF within the first year after ECV has been approximately 50% [19] with most of the recurrences occurring early after ECV [15, 16], and no change during the recent years [3, 15, 16]. The high recurrence rate of AF has been attributed to electrophysiological remodeling of the atria caused by AF [20]. It was not a surprise that the use of antiarrhythmic agents and a history of (failed) AF ablation predicted higher recurrence rate (Table 1), since they reflect high baseline AF burden in this subset of patients. Similarly, the use of digoxin was more common in those with failed ECV even if its use in rate control of AF is questionable. One unexpected finding in the present study was that low ventricular rate predicted success of ECV, and was also associated with less frequent early recurrences. The mechanisms for this finding remain unknown and need further research.

Almost half of the patients developed AF recurrence at the end of 1-month follow-up in this large real world cohort study. The composite end point combining ECV failure and early AF recurrence thereafter has been rarely analyzed in clinical studies previously, although this combined endpoint is very important from a clinical stand point. The prediction of ineffective ECV is valuable in the decision-making when considering the rationality of rhythm control strategy in an individual patient. The present analysis suggests that e.g. female patients with moderate renal failure are at high risk (~60%) for ineffective ECV. In clinical practice highly symptomatic patients with renal impairment may benefit from early consideration of AF ablation; and less symptomatic patients deferral of ECV.

This study has all the limitations of retrospective study. Notably, catheter ablation therapy was rarely used in this population. Secondly, symptomatic recurrences of AF underestimate the real recurrence rate since asymptomatic AF episodes are common in this patient population.

## Conclusions

In conclusion, for the first time we identified predictors for a clinically meaningful composite endpoint i.e. ineffective ECV. Our results may help to identify patients with unfavorable rhythm control strategy and rationalize the management of AF lasting >48 h.

## Abbreviations

AF: Atrial fibrillation
ECG: Electrocardiography
ECV: Elective cardioversion
eGFR: Estimated glomerular filtration rate
ICD: International Statistical Classification of Diseases and Related Health Problems
IQR: Inter-quartile range
MDRD: Modification of Diet in Renal Disease
NCSP: Nordic Classification of Surgical Procedures
OR: Odds ratio

## References

[1] Go AS, Hylek EM, Phillips KA, Chang Y, Henault LE, Selby JV (2001). Prevalence of diagnosed atrial fibrillation in adults: national implications for rhythm management and stroke prevention: the AnTicoagulation and Risk Factors in Atrial Fibrillation (ATRIA) Study. J Am Med Assoc.

[2] Wyse DG, Waldo AL, DiMarco JP, Domanski MJ, Rosenberg Y, Schron EB (2002). A comparison of rate control and rhythm control in patients with atrial fibrillation. N Engl J Med.

[3] Kuppahally SS, Foster E, Shoor S, Steimle AE (2009). Short-term and long-term success of electrical cardioversion in atrial fibrillation in managed care system. Int Arch Med.

[4] Fumagalli S, Boncinelli L, Bondi E, Caleri V, Gatto S, Di Bari M (2002). Does advanced age affect the immediate and long-term results of direct-current external cardioversion of atrial fibrillation?. J Am Geriatr Soc.

[5] Frick M, Frykman V, Jensen-Urstad M, Ostergren J, Rosenqvist M (2001). Factors predicting success rate and recurrence of atrial fibrillation after first electrical cardioversion in patients with persistent atrial fibrillation. Clin Cardiol.

[6] Elhendy A, Gentile F, Khandheria BK, Hammill SC, Gersh BJ, Bailey KR (2002). Predictors of unsuccessful electrical cardioversion in atrial fibrillation. Am J Cardiol.

[7] Palomäki A, Mustonen P, Hartikainen JE, Nuotio I, Kiviniemi T, Ylitalo A (2016). Strokes after cardioversion of atrial fibrillation--The FibStroke study. Int J Cardiol.

[8] Airaksinen KE, Grönberg T, Nuotio I, Nikkinen M, Ylitalo A, Biancari F (2013). Thromboembolic complications after cardioversion of acute atrial fibrillation: the FinCV (Finnish CardioVersion) study. J Am Coll Cardiol.

[9] Van Gelder IC, Crijns HJ, Van Gilst WH, Verwer R, Lie KI (1991). Prediction of uneventful cardioversion and maintenance of sinus rhythm from direct-current electrical cardioversion of chronic atrial fibrillation and flutter. Am J Cardiol.

[10] Alegret JM, Viñolas X, Sagristá J, Hernandez-Madrid A, Pérez L, Sabaté X (2007). Predictors of success and effect of biphasic energy on electrical cardioversion in patients with persistent atrial fibrillation. Europace.

[11] McCarthy C, Varghese PJ, Barritt DW (1969). Prognosis of atrial arrhythmias treated by electrical counter shock therapy. A three-year follow-up. Br Heart J.

[12] Pisters R, Nieuwlaat R, Prins MH, Le Heuzey JY, Maggioni AP, Camm AJ (2012). Clinical correlates of immediate success and outcome at 1-year follow-up of real-world cardioversion of atrial fibrillation: the Euro Heart Survey. Europace.

[13] Kirchhof P, Andresen D, Bosch R, Borggrefe M, Meinertz T, Parade U (2012). Short-term versus long-term antiarrhythmic drug treatment after cardioversion of atrial fibrillation (Flec-SL): a prospective, randomised, open-label, blinded endpoint assessment trial. Lancet.

[14] Fetsch T, Bauer P, Engberding R, Koch HP, Lukl J, Meinertz T (2004). Prevention of atrial fibrillation after cardioversion: results of the PAFAC trial. Eur Heart J.

[15] Raitt MH, Volgman AS, Zoble RG, Charbonneau L, Padder FA, O’Hara GE (2006). Prediction of the recurrence of atrial fibrillation after cardioversion in the Atrial Fibrillation Follow-up Investigation of Rhythm Management (AFFIRM) study. Am Heart J.

[16] Melduni RM, Lee HC, Bailey KR, Miller FA, Hodge DO, Seward JB (2015). Real-time physiologic biomarker for prediction of atrial fibrillation recurrence, stroke, and mortality after electrical cardioversion: A prospective observational study. Am Heart J.

[17] Disertori M, Lombardi F, Barlera S, Latini R, Maggioni AP, Zeni P (2010). Clinical predictors of atrial fibrillation recurrence in the Gruppo Italiano per lo Studio della Sopravvivenza nell’Infarto Miocardico-Atrial Fibrillation (GISSI-AF) trial. Am Heart J.

[18] Schmidt M, Daccarett M, Rittger H, Marschang H, Holzmann S, Jung P (2011). Renal dysfunction and atrial fibrillation recurrence following cardioversion. J Cardiovasc Electrophysiol.

[19] Lown B (1967). Electrical reversion of cardiac arrhythmias. Br Heart J.

[20] Allessie M, Ausma J, Schotten U (2002). Electrical, contractile and structural remodeling during atrial fibrillation. Cardiovasc Res.

